# Cancer and Atrial Fibrillation Comorbidities Among 25 Million Citizens in Shanghai, China: Medical Insurance Database Study

**DOI:** 10.2196/40149

**Published:** 2023-10-17

**Authors:** Mu Chen, Cheng Li, Jiwei Zhang, Xin Cui, Wenqi Tian, Peng Liao, Qunshan Wang, Jian Sun, Li Luo, Hong Wu, Yi-Gang Li

**Affiliations:** 1 Department of Cardiology, Xinhua Hospital School of Medicine Shanghai Jiao Tong University Shanghai China; 2 Shanghai Key Laboratory of Compound Chinese Medicines, The Ministry of Education Key Laboratory for Standardization of Chinese Medicines Institute of Chinese Materia Medica Shanghai University of Traditional Chinese Medicine Shanghai China; 3 Shanghai Health Statistics Center Shanghai China; 4 School of Public Health Fudan University Shanghai China; 5 Shanghai Municipal Health Commission Shanghai China

**Keywords:** cardiovascular, malignancy, arrhythmia, cardio-oncology, prevalence, epidemiology, atrial fibrillation

## Abstract

**Background:**

With population aging, the prevalence of both cancer and atrial fibrillation (AF) have increased. However, there is scarce epidemiological data concerning the comorbid state of cancer and AF in low- and middle-income countries, including China.

**Objective:**

We aimed to evaluate the site-, sex-, and age-specific profiles of cancer and AF comorbidities in Chinese populations.

**Methods:**

Data from the Shanghai Municipal Health Commission database between 2015 and 2020 were screened, covering all medical records of Shanghai residents with medical insurance. Site-specific cancer profiles were evaluated for the population with AF relative to the age- and sex-adjusted population of residents without AF. The sex distribution and peak age of cancer diagnosis were also assessed.

**Results:**

A total of 25,964,447 adult patients were screened. Among them, 22,185 patients presented cancers comorbid with AF (median 77, IQR 67-82 years of age; men: n=13,631, 61.44%), while 839,864 presented cancers without AF (median 67, IQR 57-72 years of age; men: n=419,020, 49.89%), thus yielding a higher cancer prevalence among residents with AF (8.27%) than among those without AF (6.05%; *P*<.001). In the population with AF, the most prevalent cancer type was lung cancer, followed by colorectal, male genital organ, stomach, breast, liver, bladder, thyroid, leukemia, and esophageal cancers. AF was associated with an average of nearly 1.4-fold (prevalence ratio [PR] 1.37, 95% CI 1.35-1.38) increased prevalence of cancer after adjusting for age and sex. For site-specific analyses, an increased prevalence of cancer in the population with AF was observed in 20 of 21 cancer sites. This increased prevalence was most prominent for nonsolid tumors, including multiple myeloma (PR 2.56, 95% CI 2.28-2.87), leukemia (PR 1.73, 95% CI 1.57-1.90), and non-Hodgkin lymphoma (PR 1.59, 95% CI 1.43-1.77); intrathoracic malignancies, including mediastinum (PR 2.34, 95% CI 1.89-2.90), lung (PR 1.64, 95% CI 1.59-1.69), and esophageal cancers (PR 1.41, 95% CI 1.28-1.56); bone and soft tissue neoplasms (PR 1.56, 95% CI 1.37-1.77); and kidney cancer (PR 1.53, 95% CI 1.36-1.72). Cancer prevalence in the population with AF relative to that in the population without AF was higher in men than in women in 14 of 18 cancer sites, and female predominance was only observed for thyroid cancer. The peak age of index cancer diagnosis was lower in the population with AF (age group: 70-74 years) than in that without AF (age group: 75-79 years), especially for specific cancer types, including thyroid, central nervous system, mediastinum, esophageal, bladder, and biliary cancers.

**Conclusions:**

Patients with AF are associated with increased prevalence, heightened male predominance, and younger peak age of cancer. Further studies are needed to determine whether early screening of specific cancers is cost-effective and beneficial for patients with AF.

## Introduction

Atrial fibrillation (AF) is the most prevalent arrhythmia and affects approximately 1% of the general population [1,2]. Besides its relation to cardiovascular complications and mortality, increased risk of various noncardiovascular diseases, including cancers, was evident in a population with AF [3]. Cancer is the leading cause of noncardiovascular deaths and is associated with increased major bleeding, intracranial hemorrhage, and arguably thromboembolic events in patients with AF [4-6]. On the other hand, new-onset AF was related to a poorer prognosis in patients with cancer [7-9]. With rapid population aging and improved survival of both diseases, AF and cancer comorbidities are becoming increasingly clinically important and prevalent and should gain more attention from both cardiologists and oncologists.

Due to the asymptomatic nature of early-stage cancer and the intermittent onset of paroxysmal AF, diagnoses of both cancer and AF may experience years of latency, especially in low- and middle-income countries (LMICs) [10,11]. Wearable devices and mobile health might largely improve the screening efficiency and early detection of both cancer and AF but are not widely adopted currently [12-14]. Consequently, the time of diagnosis of AF and cancer does not reflect the true onset time of either disease, and preclinical stages, which may involve carcinoma in situ or atrial high-rate episodes, can last years. In addition, common risk factors (eg, smoking, obesity, and aging) and related molecular pathways (eg, chronic inflammation and autonomous dysregulation) might also contribute to the causality of the co-occurrence of AF and cancer [15]. Taken together, index diagnosis of AF in patients with cancer or cancer in patients with AF based on the temporal relationship between the two disorders might contain nonnegligible selection or lead-time bias [3,5,7,8]. Such potential bias was indicated by the fact that the highest diagnosis rate of new-onset AF occurred in the first 60 days after cancer diagnosis [9]. This was attributed, at least partially, to the intensive cardiovascular examinations of newly diagnosed patients with cancer.

The prevalence of both AF and cancer, as well as site-specific cancer types, varied across different regions, races, and ethnicities [16]. Consequently, the prevalence of AF-cancer comorbidities should also present with regional, racial, and ethnic differences. There is scarce epidemiological data concerning the comorbid state of cancer and AF, especially in LMICs, including China. Taking advantage of the government-issued database containing all sources of medical records from 25 million Shanghai citizens [1], this study investigated the site-, sex-, and age-specific profiles of comorbid cancer in the population with AF in Shanghai, thus providing insights regarding cancer and AF comorbidities in the Chinese population.

## Methods

### Database and Study Population

Data were retrieved from the Shanghai Municipal Health Commission database from 2015 to 2020 [1]. The database was administered by the Shanghai municipal government and covered all sources of medical records in Shanghai from 2346 medical institutions, including information on inpatient and outpatient visits at all hospitals and clinics and routine primary and family care. With health insurance coverage of over 96%, the local population with medical insurance largely represents the residents of Shanghai [1]. The government-instituted universal health insurance included the Urban Residents’ Basic Medical Insurance scheme, the Urban Employee Basic Medical Insurance scheme, and the New Rural Cooperative Medical scheme. Patients without local medical insurance were excluded to avoid the inclusion of nonlocal patients who sought temporary medical services in Shanghai. Pediatric patients were also excluded. Adult patients (aged ≥20 years) in the database were screened for cancer and AF. Patients with cancer and AF comorbidities were defined as the patients with index diagnoses of cancer and AF between 2015 and 2020, regardless of the temporal order of the index date between the two diagnoses.

Diagnoses were recorded using the *International Statistical Classification of Diseases, Tenth Revision* (*ICD-10*). AF was identified by *ICD-10* codes I48.01-03 and O99.418. Cancers in general (*ICD-10* codes: C00-C97) and of individual systems were screened in patients with and without AF. A total of 21 types of cancer were listed as follows: esophageal cancer (C15), stomach cancer (C16), colorectal cancer (C18 to C21), liver cancer (C22), biliary cancer (C23 and C24), pancreatic cancer (C25), head and neck cancer (C00 to C14 and C30 to C32), lung cancer (C34), malignant neoplasm of mediastinum (C37 and C38), malignant neoplasm of bone and soft tissue (C40, C41, and C45 to C49), melanoma (C43), breast cancer (C50), gynecologic cancer (C51 to C58), male genital cancer (C60 to C63), renal cancer (C64), bladder cancer (C67), central nervous system (CNS) cancer (C69 to C72), thyroid cancer (C73), non-Hodgkin lymphoma (C82 to C85), multiple myeloma (C90), and leukemia (C91 to C95). Carcinoma in situ (D00 to D09) and borderline tumors (D37 to D48) were not included. The detailed *ICD-10* codes are listed in Table S1 in Multimedia Appendix 1. For analyses of sex disparities, gynecologic, breast, and male genital organ cancers were not included. China has released a nationwide regulation for cancer registration, which requires health care systems to report cancer cases since 2015 [17]. Therefore, cancer diagnosis data in this study was assumed to be reliable because the Chinese government provides financial support for patients with cancer-related *ICD-10* codes on the basis of clinical pathologic assessments.

### Statistical Analysis

Data are presented as median (IQR) or as absolute values and percentages. The prevalence of comorbid cancer in the population with AF was calculated from the number of patients with cancer and AF divided by the total number of patients with AF. Due to different age and sex distributions between populations with and without AF, standardization was conducted for patients without AF according to the age and sex distributions of patients with AF (the numbers of patients before and after the adjustment are shown in Table S2 in Multimedia Appendix 1). The prevalence of cancer in patients without AF was subsequently calculated from the number of sex- and age-adjusted patients without AF who had cancer divided by the total number of sex- and age-adjusted patients without AF. The sex-specific cancer prevalence in patients with and without AF was also analyzed and compared. Pearson chi-square tests or Fisher precision probability tests were used to analyze the differences in the prevalence of various cancer types between populations with and without AF. The relative prevalence of cancer was presented as the prevalence ratio (PR) and 95% CI. *P*<.05 was considered statistically significant. All analyses were performed with SPSS 22.0 (IBM Corp).

### Ethical Considerations

This study was reviewed and approved by the ethics committee of Xinhua Hospital, School of Medicine, Shanghai Jiao Tong University (XHEC-D-2022-043). Informed consent was exempted due to the retrospective design and the anonymized and deidentified patient records.

## Results

### Baseline Characteristics

A total of 25,964,447 adult patients (median 47, IQR 32-62 years of age; men: n=12,649,586, 48.72%) were included in the analyses (Table S3 in Multimedia Appendix 1). Among them, 22,185 patients presented with comorbid AF and cancer (median 77, IQR 67-82 years of age; men: n=13,631, 61.44%). Cancers were found in 839,864 individuals without AF (median 67, IQR 57-72 years of age; men: n=419,020, 49.89%), while AF was observed in 246,216 patients without cancer (median 77, IQR 67-87 years of age; men: n=118,063, 47.95%). As shown in Table 1, despite a uniformly higher prevalence of cancer in patients with AF, the top 10 cancers in the populations with and without AF were essentially identical, with the exceptions of leukemia in the population with AF and pancreatic cancer in the population without AF. The most prevalent cancer in the population with AF was lung cancer, followed by colorectal, male genital organ, stomach, breast, liver, bladder, thyroid, leukemia, and esophageal cancers.

**Table 1:** Prevalence of different cancers in patients with and without atrial fibrillation (AF).

Cancer site	Patients with AF (n=268,401), n (%)	Patients without AF (n=3,954,796^a^), n (%)
Lung	4572 (1.70)	41,168 (1.04)
Colorectum	2389 (0.89)	27,976 (0.71)
Male genital organs	1588 (0.59)	21,093 (0.53)
Stomach	1529 (0.57)	17,982 (0.45)
Breast	1032 (0.38)	12,427 (0.31)
Liver	774 (0.29)	8247 (0.21)
Bladder	593 (0.22)	7313 (0.18)
Thyroid	565 (0.21)	6927 (0.18)
Leukemia	473 (0.18)	4037 (0.10)
Esophagus	438 (0.16)	4570 (0.12)
Pancreas	424 (0.16)	5359 (0.14)

^a^Data from patients without AF were age- and sex-adjusted.

### Increased Prevalence of Cancer in Patients With AF

**Figure 1:**
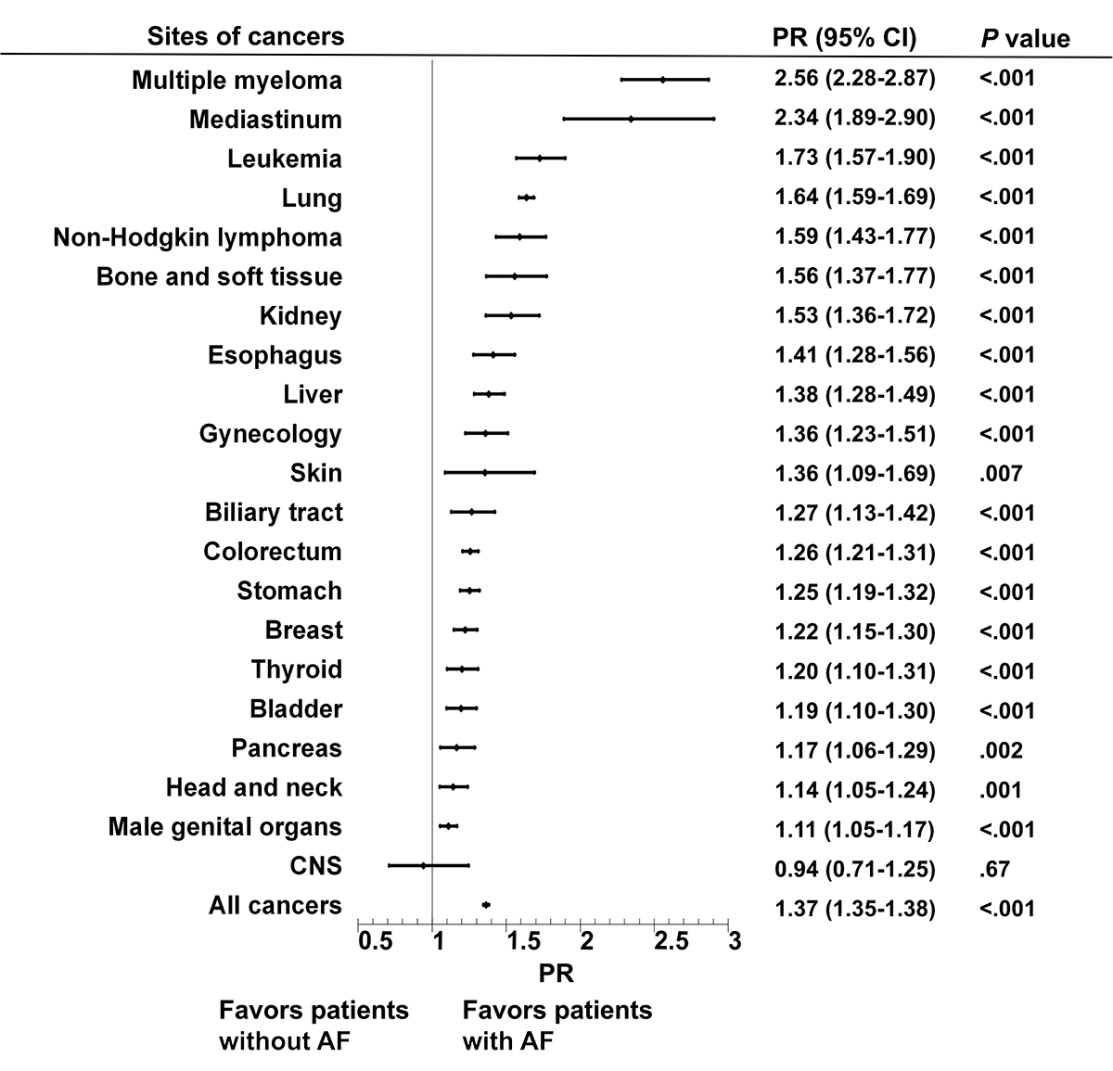
Increased cancer prevalence in patients with AF. Plot showing relative prevalence of site-specific cancers in patients with and without AF, ordered from largest to smallest relative prevalence. AF: atrial fibrillation; CNS: central nervous system; PR: prevalence ratio.

As shown in Table 2, the prevalence of cancer in patients with AF (8.27% in total; 10.35% in men and 6.26% in women) was higher than that in patients without AF (6.05% in total; 7.21% in men and 4.89% in women; *P*<.001 for all comparisons). AF was associated with a nearly 1.4-fold higher prevalence of cancer after adjusting for age and sex (PR 1.37, 95% CI 1.35-1.38; Figure 1). A consistently increased prevalence of comorbidity with AF was observed in 20 of 21 cancer sites, with the only exception being CNS cancers (PR 0.94, 95% CI 0.71-1.25). The strongest association was observed in multiple myeloma (PR 2.56, 95% CI 2.28-2.87), followed by mediastinum cancer, leukemia, lung cancer, bone and soft tissue neoplasms, kidney cancer, and esophageal cancer.

**Table 2:** Prevalence of different cancers in men and women with and without atrial fibrillation (AF).

Cancer site	All patients				Men				Women			
	With AF, %	Without AF^a^, %	Chi-square	*P* value^b^	With AF, %	Without AF, %	Chi-square	*P* value	With AF, %	Without AF, %	Chi-square	*P* value
All sites	8.27	6.05	2118.27	<.001	10.35	7.21	1785.11	<.001	6.26	4.89	506.75	<.001
Head and neck	0.23	0.20	10.21	.001	0.32	0.28	6.99	.008	0.14	0.12	3.53	.06
Esophagus	0.16	0.12	48.15	<.001	0.23	0.15	42.32	<.001	0.10	0.08	8.27	.004
Stomach	0.57	0.45	72.26	<.001	0.78	0.60	66.45	<.001	0.36	0.31	11.07	.001
Colorectum	0.89	0.71	117.52	<.001	1.10	0.82	114.96	<.001	0.69	0.60	17.76	<.001
Liver	0.29	0.21	75.17	<.001	0.38	0.27	61.96	<.001	0.20	0.15	16.01	.001
Biliary tract	0.12	0.09	16.39	.001	0.11	0.09	9.43	.002	0.12	0.10	7.15	.008
Pancreas	0.16	0.14	9.28	.002	0.16	0.14	2.04	.15	0.16	0.13	8.92	.003
Lung	1.70	1.04	1029.57	<.001	2.33	1.28	1010.04	<.001	1.10	0.81	138.10	<.001
Mediastinum	0.04	0.02	64.44	<.001	0.05	0.02	47.38	<.001	0.03	0.01	18.55	<.001
Bone and soft tissue	0.09	0.06	45.43	<.001	0.11	0.07	29.22	<.001	0.08	0.05	17.45	<.001
Skin	0.03	0.02	7.39	.007	0.03	0.02	6.08	.01	0.03	0.02	1.99	.16
Breast	0.38	0.31	39.07	<.001	0.02	0.05	17.51	<.001	0.73	0.57	58.82	<.001
Kidney	0.12	0.08	52.26	<.001	0.16	0.10	38.95	<.001	0.07	0.05	14.61	.001
Bladder	0.22	0.18	17.46	<.001	0.36	0.29	22.26	<.001	0.09	0.09	0.06	.81
Gynecology	0.14	0.10	25.16	<.001	N/A^c^	N/A	N/A	N/A	0.28	0.21	25.16	<.001
Male genital organs	0.59	0.53	15.99	.001	1.21	1.02	40.54	<.001	N/A	N/A	N/A	N/A
Thyroid	0.21	0.18	17.74	<.001	0.13	0.11	2.23	.14	0.29	0.24	16.55	<.001
Non-Hodgkin lymphoma	0.14	0.09	76.72	<.001	0.17	0.11	44.94	<.001	0.12	0.07	32.93	<.001
Multiple myeloma	0.13	0.05	277.42	<.001	0.16	0.06	202.11	<.001	0.09	0.04	82.89	<.001
Leukemia	0.18	0.10	129.55	<.001	0.21	0.12	84.64	<.001	0.14	0.08	45.90	<.001
CNS^d^	0.02	0.02	0.18	.67	0.03	0.02	1.00	.32	0.01	0.02	2.73	.10

^a^Data from patients without AF were age-adjusted.

^b^*P*<.05 was considered statistically significant.

^c^N/A: not applicable.

^d^CNS: central nervous system.

### Sex Disparities in Cancer Prevalence

The increased prevalence of various cancer types in the population with AF was further analyzed regarding sex. As shown in Table 3, the strongest association between AF and cancer was found in multiple myeloma (in men: PR 2.73, 95% CI 2.36-3.16; in women: PR 2.32, 95% CI 1.93-2.80) and mediastinum cancer (in men: PR 2.50, 95% CI 1.91-3.28; in women: PR 2.12, 95% CI 1.49-3.01) for both sexes. The increased AF prevalence in patients with lung cancer ranked third in men and seventh in women. The increased AF prevalence in patients with gynecologic cancer ranked eighth in women.

**Table 3:** Atrial fibrillation (AF)–related cancer prevalence in men and women.

Cancer site	Men		Women	
	PR^a^ (95% CI)	*P* value	PR (95% CI)	*P* value
Multiple myeloma	2.73 (2.36-3.16)	<.001	2.32 (1.93-2.80)	<.001
Mediastinum	2.50 (1.91-3.28)	<.001	2.12 (1.49-3.01)	<.001
Lung	1.81 (1.75-1.88)	<.001	1.37 (1.30-1.44)	<.001
Leukemia	1.77 (1.57-2.00)	<.001	1.66 (1.43-1.93)	<.001
Non-Hodgkin lymphoma	1.59 (1.39-1.83)	<.001	1.60 (1.36-1.88)	<.001
Bone and soft tissue	1.59 (1.34-1.88)	<.001	1.53 (1.25-1.87)	<.001
Kidney	1.56 (1.36-1.80)	<.001	1.49 (1.21-1.83)	<.001
Esophagus	1.48 (1.31-1.67)	<.001	1.29 (1.08-1.53)	.004
Skin	1.47 (1.08-1.99)	.01	1.26 (0.91-1.73)	.16
Liver	1.44 (1.31-1.57)	<.001	1.29 (1.14-1.46)	<.001

^a^PR: prevalence ratio.

**Figure 2:**
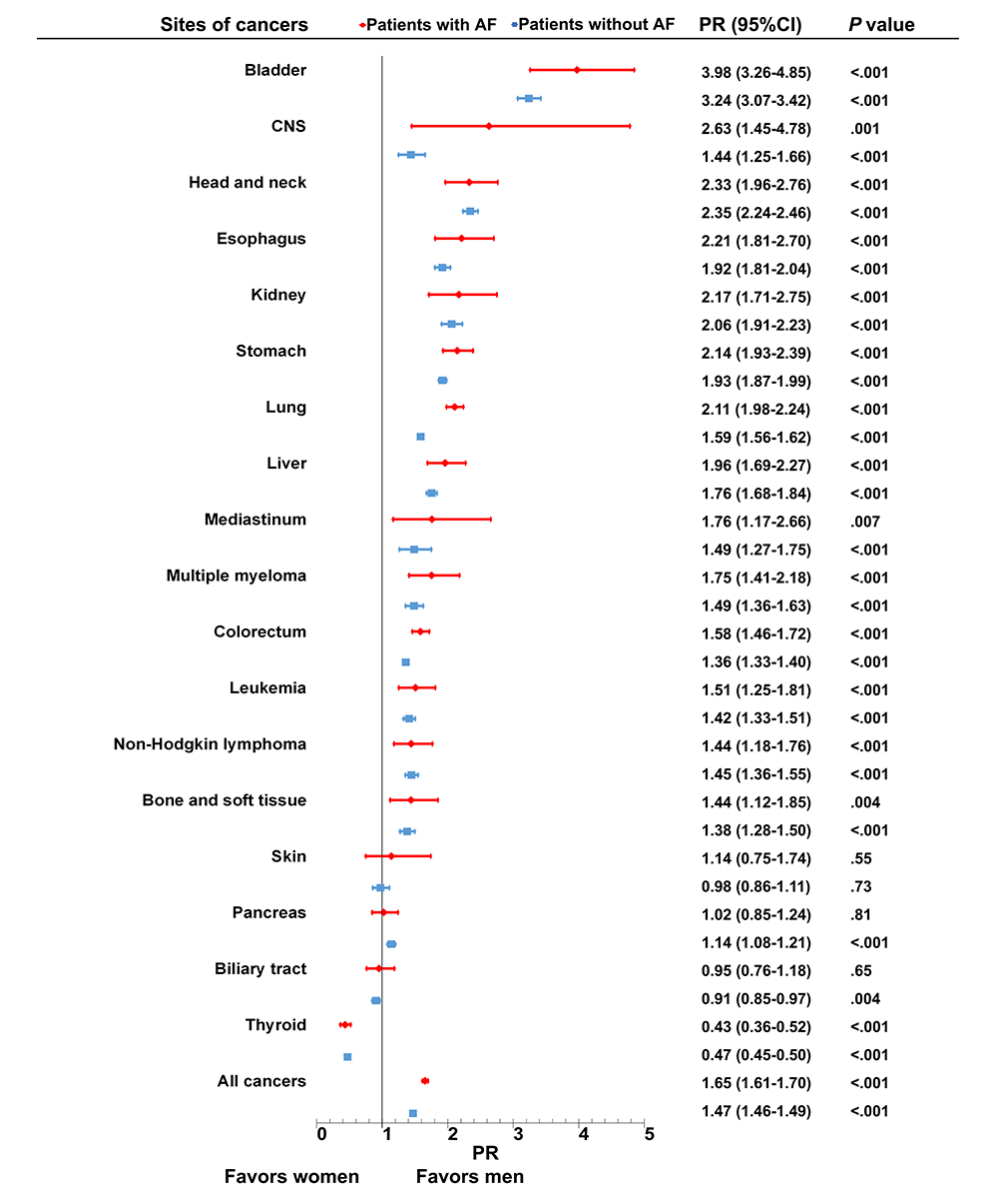
Sex disparities in cancer prevalence among different cancer types in patients with and without AF. Plot showing relative prevalence of site-specific cancers comparing men to women among patients with (red) and without (blue) AF. The cancer types are ranked from largest to smallest relative risk in patients with AF. A total of 18 cancer sites were analyzed. Gynecologic cancer, breast cancer, and cancer of male genital organs (mainly prostate cancer) were not included in the analyses for sex disparities. AF: atrial fibrillation; CNS: central nervous system; PR: prevalence ratio.

As shown in Figure 2, cancer prevalence showed a male predominance in the population without AF (PR 1.47, 95% CI 1.46-1.49), which was further strengthened in the population with AF (PR 1.65, 95% CI 1.61-1.70). In the population with AF, the prevalence of cancer was higher in men than in women at 14 of 18 cancer sites (gynecologic, breast, and male genital organ cancers were excluded). Among them, a male predominance of over a 2:1 male to female ratio was found in bladder, CNS, head and neck, esophageal, kidney, stomach, and lung cancers. Cancer risk in the population with AF was similar for skin, pancreas, and biliary cancers between sexes, while a female predominance was found in thyroid cancer.

### Peak Age of Cancer Diagnosis in Patients With AF

**Figure 3:**
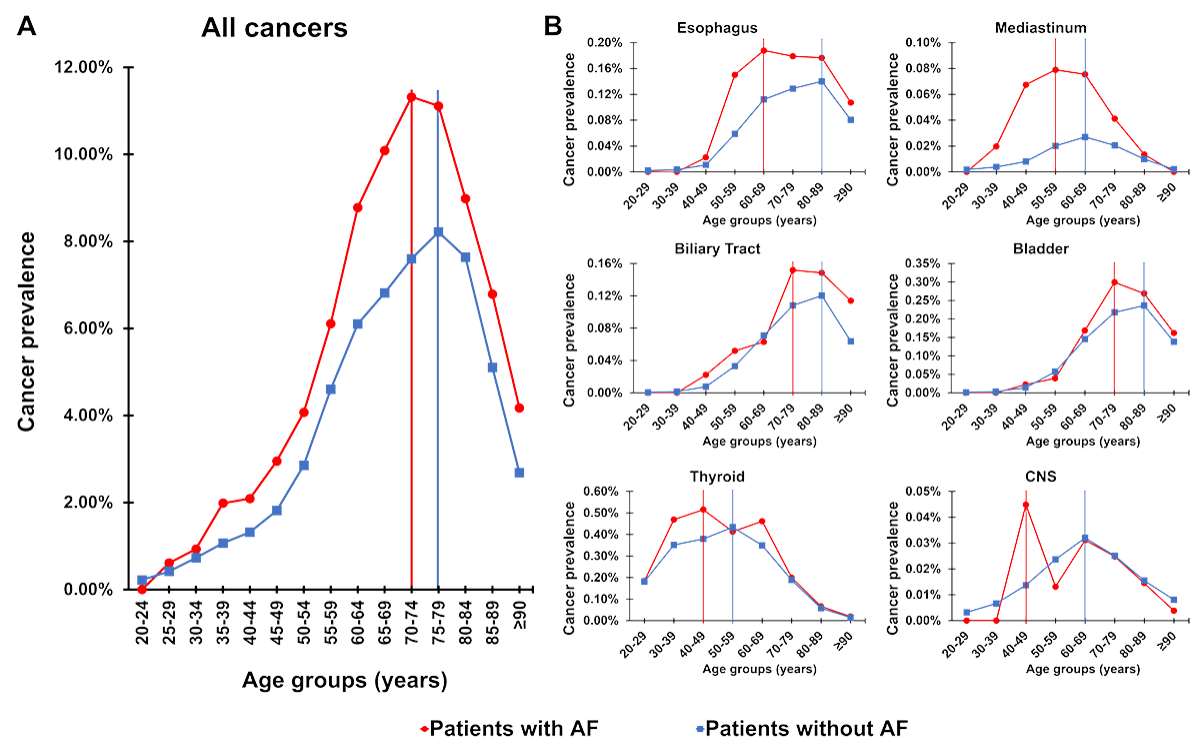
Peak age of cancer in general (A) and specific cancer types (B) in patients with and without AF. AF: atrial fibrillation; CNS: central nervous system.

Overall, there was a younger peak age of cancer diagnosis in the population with AF (range 70-74 years) than in that without AF (range 75-79 years; Figure 3A). For specific cancer types, a younger peak age of cancer diagnosis was observed in the population with AF than in that without AF, including thyroid (with AF: range 40-49 years; without AF: range 50-59 years), CNS (with AF: range 40-49 years; without AF: range 60-69 years), mediastinum (with AF: range 50-59 years; without AF: range 60-69 years), esophageal (with AF: range 60-69 years; without AF: range 80-89 years), bladder (with AF: range 70-79 years; without AF: range 80-89 years), and biliary (with AF: range 70-79 years; without AF: range 80-89 years) cancers (Figure 3B). In other types of cancers, the peak age of cancer diagnosis was similar between the populations with and without AF (Figure S1 in Multimedia Appendix 1).

## Discussion

### Main Findings

In this whole population–based medical insurance database study, we reported the prevalence of the AF-cancer comorbid state in Shanghai, the largest city in China. The main findings were as follows: (1) the most prevalent cancer types were essentially identical between the populations with and without AF; (2) cancer prevalence was higher in 20 of 21 cancer types in patients with AF compared to those without AF; (3) prevalence of nonsolid tumors, intrathoracic malignancies, bone and soft tissue cancers, and kidney cancer were ≥1.4-fold higher in the population with AF; (4) male predominance of cancer was more prominent in the population with AF; and (5) a younger peak age of the index cancer diagnosis was observed in the population with AF, especially for esophageal, mediastinum, thyroid, bladder, biliary, and CNS cancers.

### Increased Prevalence of Cancer With Comorbid AF

Increasingly, AF has been recognized as an important comorbidity in cancer. Previous studies reported that patients with cancer have an increased risk of new-onset AF compared with the general population or individuals without cancer [7,8,18]. In addition, patients with cancer and AF showed increased risks of thromboembolism, bleeding, heart failure, and mortality, which highlighted the importance of detecting and managing AF in patients with cancer [19,20]. Notably, elevated breast cancer risk was reported in patients with pre-existing AF [3,9]; however, it remained unknown if the results of these studies could be extended to other cancer types among patients with AF. This study has contributed to filling in this knowledge gap, especially in the Chinese population. Significantly increased cancer prevalence was found in the population with AF, most prominently for hematological tumors (including multiple myeloma, leukemia, and non-Hodgkin lymphoma), intrathoracic malignancies (including mediastinum, lung, and esophageal cancers), and bone, soft tissue, and kidney cancers. It is important to note that the prominent association between AF and CNS cancer, which was previously reported [8], was not observed in our study. The reason for this disparity was not clear. It is possible that the previously reported new-onset AF after CNS cancer diagnosis was mainly attributed to cardiotoxicity following cancer treatment, rather than the common pathway between oncogenesis and arrhythmogenesis.

### Challenges in Establishing Temporal Relationships Between AF and Cancer

In this study, the temporal relationship between the two diseases was not differentiated for 4 reasons. First, the diagnoses of both AF and cancer commonly present latency following the development of the disease [10,11]. Years of delay in AF diagnoses have been noticed in more than 30% of patients with prior cryptogenic stroke, even under frequent electrocardiographic screening [10]. Likewise, early diagnosis of cancer relies on screening at its asymptomatic stage, which is more challenging in LMICs due to weaker health care and economic infrastructure [21]. Therefore, it would be challenging to determine the temporal order of the index diagnoses of AF and cancer.

Second, both diseases at their preclinical stage may already predispose similar pathological effects prior to their clinical stage. Atrial high-rate episodes, the precursor to AF associated with subsequent clinically documented AF, could already present AF-prone thromboembolic, cardiovascular, and mortality risks [22]. Carcinoma in situ, the precursor to invasive cancer, may have already created a tumorous microenvironment and triggered a systemic immune response during its transition [23]. Therefore, the index diagnoses might be unable to determine the potential interconnection of the two entities at their subclinical stages.

Third, various common risk factors suggested that temporal sequencing did not necessarily imply causation. Common risk factors included aging, hypertension, diabetes, obesity, smoking, consumption of alcohol, air pollution, mental and physical stress, sleep disturbances, and westernized diet and lifestyle, among others. These risk factors led to pathological processes that precipitated both diseases, such as chronic inflammation, oxidative stress, electrolyte-handling abnormalities, and autonomous dysregulation [15]. Therefore, the AF-cancer comorbid state may comprehensively reflect the true nature of the development of the two diseases.

Finally, the temporal association may result in selection bias and lead-time bias [3,5,7,8]. The highest rate of new-onset AF in the first 60 or 90 days after cancer diagnosis may partially be attributed to frequent clinical visits, electrophysiological monitoring, and comprehensive cardiovascular evaluation that may affect cancer therapy [3,9]. On the other hand, the initiation of anticoagulation after AF diagnosis may reveal occult cancers by the alerting sign of bleeding, especially at gastrointestinal and genitourinary sites [24]. Nevertheless, a definite timeline between the onset of cancer and AF did exist in several circumstances; these conditions were commonly correlated with antineoplastic therapies, including chemotherapy, targeted therapy, surgery, and radiation, as well as cancer-related pain and stress [25]. However, the effects of arrhythmogenic cardiotoxicity differed among individuals. Those with proarrhythmic atrial substrate may be more vulnerable to AF during cancer therapy.

### Heightened Male Predominance of Cancer Among Patients With AF

Robust sex differences exist in almost all aspects of cancer and AF, including epidemiology, progression, treatment response, and survival [26-28]. A striking male predominance in cancers was found in the general population from over 60 countries [26]. Similarly, a 3:2 male to female ratio in AF prevalence was also present, despite a female predominance in the older population [27]. Due to the higher prevalence of both cancer and AF in men, it seemed reasonable to expect higher rates of the comorbid conditions in men than in women. Our results in the population without AF demonstrated a male predominance of cancer at 15 of 18 sites, which was consistent with a previous study [26]. Of note, the male to female ratio in cancer was further intensified in the population with AF. The extents to which sex differences among cancer types were strengthened by AF were not uniform among the different cancer types and were most prominent in lung and colorectal cancers. The varied extents suggested that mechanisms other than simple pile-up effects were at play. The response of sex differentiated cardiotoxicity to cancer therapy may also contribute to a heightened sex disparity in AF development [28].

### Importance of Cancer Screening in Patients With AF

As one of the most important topics in oncology, cancer screening aims to achieve early cancer detection, improve clinical outcomes, and reduce the burden of health economics. The benefits of screening for a particular cancer must outweigh the associated costs and risks of screening. The success of a screening program also depends on the disease burden, the reliability of the test, population participation, adequate resources, and staffing. Therefore, selection of the high-risk population and the cut-off age is of high importance in cancer screening, especially in LMICs compared to countries with ample resources [29]. The current cancer screening guidelines highlighted several high-risk conditions that benefited from early screening and intensive precursor surveillance for specific cancers, such as smoking for lung cancer, human papillomavirus infection for cervix cancer, family history of malignancies, and so on [30]. The results of our study suggest not only a higher prevalence but also a younger peak age of cancer diagnosis in the population with AF. Specifically, nonsolid tumors (including multiple myeloma, leukemia, and non-Hodgkin lymphoma) and intrathoracic malignancies (including lung, esophageal, and mediastinum cancers) were characterized by higher absolute prevalence, younger peak age, and stronger associations with AF than other cancer types. Therefore, early screening of nonsolid and intrathoracic cancers via methods such as complete blood count, endoscopy, and chest computed tomography may be beneficial for patients with AF. Accordingly, more intensive heart rhythm monitoring for potential AF should also be considered in these types of cancers to optimize the risk assessment and standardize anticoagulation management.

### Limitations

First, only adult patients were included in this study. AF is rare in children, and pediatric malignancies may need to be seen as a distinct subset from adult cancers due to different cancer genetics, distributions, risk factors, comorbidities, and consequently, pathophysiological mechanisms. Second, the generalizability of our findings may be limited with regard to population and time, despite the large volume of data. The profiles of both cancer and AF were undergoing a transition in China due to the constantly changing demographic structure and health economic background and were affected by other diseases and different lifestyles [31]. Shanghai, as the most industrialized region in China with the oldest population [32], may have heavier burdens of both cancer and AF than other regions in China and other LMICs [16,33]. However, the epidemiological features, medical resources, and disease management provisions in Shanghai may share a mixture of characteristics with those in LMICs and high-income countries. This may represent a future trend in other parts of China and other LMICs around the world. Third, asymptomatic AF may have gone underdiagnosed. However, thanks to the popularity of contracted family doctor services, residents in Shanghai undergo routine cardiac auscultation and intermittent handheld electrocardiogram recording, which may have largely unveiled silent AF during the 6-year inclusion period. Lastly, endogeneity bias might exist since other diseases and lifestyles also participate in the AF-cancer association.

### Conclusions

This large medical insurance database study demonstrates that the comorbid state of AF and cancer is substantial in Shanghai, China. Patients with AF face increased prevalence, heightened male predominance, and a younger peak age of cancer. Physicians and patients should be aware of the cancer risks when AF is diagnosed. Screening for AF-associated cancers, such as by complete blood count for hematological tumors or thoracic computer tomography for intrathoracic malignancies, might be considered in patients with AF after a consultation with a cardio-oncologist. Likewise, more intensive heart rhythm monitoring for potential AF might be performed in specific cancers to optimize the prognosis, assessment, and prevention of cardiovascular complications. Further studies are needed to determine whether early screening of specific cancers in patients with AF is cost-effective and beneficial. Investigations regarding the value of wearable devices and mobile health in the screening and early detection of AF-cancer comorbidity are also warranted.

## Appendix

Multimedia Appendix 1 Supplementary materials.

## Abbreviations

AF: atrial fibrillation
CNS: central nervous system
ICD-10: International Statistical Classification of Diseases, Tenth Revision
LMIC: low- and middle-income countries
PR: prevalence ratio

## Data Availability

The data that support the findings of this study were obtained from the Shanghai Municipal Health Commission. Data sets are available from the corresponding author with the permission of the Shanghai Municipal Health Commission.
