# Supplementary material for: Cancer and Atrial Fibrillation Comorbidities Among 25 Million Citizens in Shanghai, China: Medical Insurance Database Study
Source: JMIR Public Health Surveill. 2023 Oct 17;9:e40149. doi: 10.2196/40149 (PMC10618890; doi:10.2196/40149)
Supplement: Multimedia Appendix 1 [file publichealth_v9i1e40149_app1.docx]

**Table S1. ICD_10 codes for AF and cancers.**

| Category of diseases | ICD_10 codes |
| --- | --- |
| Atrial fibrillation | I48__01; I48__02; I48__03; O99.418 |
|  |  |
| Cancers | C00-C97 |
| Sites of cancers |  |
| Head and neck | C00.001; C00.901; C00.101; C01__01; C02.001; C02.101; C02.902; C02.901; C02.201; C03.001; C03.101; C03.901; C03.902; C03.903; C04.901; C05.001; C05.901; C05.101; C06.001; C06.201; C06.003; C06.002; C07__02; C07__01; C08.001; C08.101; C08.901; C08.801; C08.102; C09.801; C09.101; C09.901; C10.301; C10.901; C10.401; C11.301; C11.901; C11.101; C11.201; C11.902; C12__01; C13.901; C13.001; C14.001; C14.002; C14.003; C14.101; C14.805; C14.802; C14.803; C14.804; C14.801  C30.001; C30.002; C30.003; C30.102; C30.005; C30.101; C30.004; C39.802; C39.801  C31.001; C31.002; C31.101; C31.901; C31.201; C31.301; C31.801; C31.102  C32.001; C32.002; C32.902; C32.901; C32.101 |
| Esophagus | C15.001; C15.101; C15.201; C15.301; C15.301; C15.401; C15.902; C15.501; C15.501; C15.801; C15.802; C15.901; C15.401 |
| Stomach | C16.901; C16.802; C16.903; C16.904; C16.907; C16.804; C16.805; C16.905; C16.906; C16.001; C16.002; C16.003; C16.101; C16.201; C16.301; C16.401; C16.402; C16.501; C16.601; C16.801; C16.902 |
| Colorectum | C18.904; C18.803; C18.804; C18.901; C18.902; C18.903; C18.001; C18.002; C18.101; C18.201; C18.301; C18.401; C18.501; C18.601; C18.701; C18.702; C18.802; C19__02; C19__01; C20__01; C20__03; C20__02; C21.001; C21.801; C21.802; C21.101; C26.803 |
| Liver | C22.001; C22.002; C22.101; C22.902; C22.301; C22.901; C22.102 |
| Biliary tract | C23__01; C24.001; C24.002; C24.003; C24.004; C24.005; C24.006; C24.102; C24.803; C24.801; C24.802; C24.101 |
| Pancreas | C25.402; C25.801; C25.901; C25.902; C25.401; C25.101; C25.201; C25.301; C25.001 |
| Lung | C34.001; C34.906; C34.102; C34.201; C34.301; C34.302; C34.801; C34.802; C34.901; C34.902; C34.903; C34.904; C34.905; C34.002; C34.101 |
| Mediastinum | C37__02; C37__01  C38.001; C38.002; C38.101; C38.401; C38.301; C38.302; C38.201 |
| Bone and soft tissue | C40.001; C40.002; C40.101; C40.201; C40.302; C40.203; C40.301; C40.901; C40.003; C40.202; C41.001; C41.205; C41.003; C41.101; C41.102; C41.201; C41.301; C41.302; C41.401; C41.402; C41.405; C41.404; C41.202; C41.203; C41.204; C41.002  C45.001; C45.101; C45.102; C45.702; C45.701; C45.201; C46.001; C46.301; C46.201; C46.101; C47.901; C47.001; C47.301; C48.001; C48.002; C48.003; C48.004; C48.202; C48.101; C48.102; C48.201; C48.005; C49.001; C49.903; C49.003; C49.004; C49.005; C49.101; C49.102; C49.201; C49.202; C49.301; C49.302; C49.303; C49.304; C49.401; C49.501; C49.502; C49.503; C49.601; C49.602; C49.901; C49.506; C49.006; C49.402; C49.504; C49.902; C49.002 |
| Skin | C43.001; C43.903; C43.201; C43.301; C43.401; C43.501; C43.502; C43.601; C43.602; C43.701; C43.901; C43.702; C43.703; C43.801; C43.302; C43.402; C43.902; C43.101 |
| Breast | C50.001; C50.101; C50.201; C50.301; C50.401; C50.501; C50.002; C50.901; C50.902; C50.903; C50.904; C50.905; C50.-; C50.601 |
| Gynecology | C51.001; C51.101; C51.902; C51.801; C51.901; C51.201  C52__02; C52__01  C53.001; C53.903; C53.902; C53.901  C54.102; C54.901; C54.101; C54.103; C54.001; C54.104; C55__02; C55__01  C56__01; C56__02; C56__07; C56__04; C56__06; C56__03; C56__05  C57.001; C57.002; C57.101; C57.901; C57.401; C57.804; C57.301  C58__01; C58__02; C58__03; C58.001; C58__05; C58__06; C58__07; C58__04 |
| Male genital organs | C60.001; C60.901; C60.201; C60.101; C61__02; C61__01; C62.901; C62.001; C63.001; C63.801; C63.701; C63.201 |
| Kidney | C64__02; C64__01 |
| Bladder | C67.001; C67.101; C67.201; C67.301; C67.902; C67.501; C67.701; C67.901; C67.401 |
| CNS | C69.001; C69.101; C69.201; C69.301; C69.901; C69.402; C69.501; C69.502; C69.601; C69.401; C71.301; C71.401; C71.501; C71.502; C71.601; C71.701; C71.702; C71.801; C71.802; C71.803; C71.804; C71.805; C71.806; C71.901; C71.902; C71.903; C71.003; C71.004; C71.-; C71.001; C71.002; C71.101; C71.102; C71.201; C70.001; C70.901; C70.101; C72.001; C72.002; C72.101; C72.501; C72.301; C72.901; C72.902; C72.201 |
| Thyroid | C73__01; C73__02; C73__05; C73__04; C73__06; C73__03 |
| Non-Hodgkin's lymphoma | C82.101; C82.102; C82.902; C82.701; C82.901; C83.201; C83.301; C83.401; C83.402; C83.302; C83.801; C83.001; C83.303; C83.202; C83.701; C84.001; C84.001; C84.101; C84.501; C84.201; C84.301; C84.401; C84.101; C85.002; C85.926; C85.004; C85.005; C85.101; C85.902; C85.903; C85.904; C85.905; C85.906; C85.907; C85.908; C85.909; C85.910; C85.911; C85.912; C85.913; C85.914; C85.915; C85.916; C85.917; C85.918; C85.919; C85.920; C85.921; C85.927; C85.928; C85.929; C85.930; C85.931; C85.932; C85.933; C85.934; C85.935; C85.936; C85.937; C85.938; C85.939; C85.940; C85.006; C85.007; C85.008; C85.011; C85.012; C85.013; C85.014; C85.016; C85.017; C85.701; C85.922; C85.923; C85.924; C85.925; C85.003 |
| Multiple myeloma | C90.001; C90.002; C90.003+; C90.203; C90.101; C90.201; C90.202; C90.004+ |
| Leukemia | C91.001; C91.002; C91.101; C91.201; C91.301; C91.701; C91.501; C91.901; C91.005; C91.003; C91.004; C91.401; C92.001; C92.003; C92.101; C92.102; C92.103; C92.201; C92.301; C92.401; C92.501; C92.701; C92.702; C92.703; C92.704; C92.004; C92.002; C93.001; C93.901; C93.201; C93.101; C94.001; C94.002; C94.003; C94.101; C94.701; C94.201; C94.301; C94.401; C94.501; C94.102; C95.001; C95.002; C95.101; C95.201; C95.904+; C95.901; C95.902+; C95.003; C95.004; C95.701 |

**Table S2 The numbers of patients before and after the adjustment.**

|  | AF patients | | | Non-AF subjects | | | | | |
| --- | --- | --- | --- | --- | --- | --- | --- | --- | --- |
|  | Crude patient numbers | | | Crude patient numbers | | | The numbers of patients after the adjustment | | |
|  | Men | Women | All | Men | Women | All | Men* | Women* | All § |
| Total | 131694 | 136707 | 268401 | 12781280 | 13451569 | 26232849 | 2062302 | 1932945 | 3954796 |
| Sites of cancers |  |  |  |  |  |  |  |  |  |
| All cancers | 13631 | 8554 | 22185 | 419020 | 420844 | 839864 | 148646 | 94464 | 239192 |
| Head and neck | 426 | 190 | 615 | 23118 | 12119 | 35237 | 5844 | 2332 | 7929 |
| Esophagus | 298 | 140 | 438 | 8577 | 3702 | 12279 | 3155 | 1537 | 4570 |
| Stomach | 1031 | 499 | 1529 | 32251 | 18910 | 51161 | 12416 | 6045 | 17982 |
| Colorectum | 1443 | 946 | 2389 | 43338 | 33397 | 76735 | 16878 | 11606 | 27976 |
| Liver | 506 | 268 | 774 | 17346 | 8146 | 25492 | 5511 | 2938 | 8247 |
| Biliary tract | 150 | 164 | 314 | 4482 | 4733 | 9215 | 1811 | 1866 | 3649 |
| Pancreas | 211 | 214 | 424 | 8136 | 6667 | 14803 | 2985 | 2447 | 5359 |
| Lung | 3062 | 1510 | 4572 | 76063 | 69888 | 145951 | 26480 | 15595 | 41168 |
| Mediastinum | 61 | 36 | 97 | 1679 | 1527 | 3207 | 382 | 240 | 610 |
| Bone and soft tissue | 147 | 106 | 252 | 5215 | 4704 | 9919 | 1448 | 981 | 2385 |
| Skin | 45 | 41 | 86 | 1529 | 1893 | 3423 | 481 | 461 | 934 |
| Breast | 29 | 1004 | 1032 | 2898 | 62257 | 65156 | 980 | 11040 | 12427 |
| Kidney | 211 | 101 | 311 | 7545 | 4021 | 11566 | 2114 | 960 | 2989 |
| Bladder | 471 | 123 | 593 | 13090 | 4283 | 17373 | 5889 | 1701 | 7313 |
| Gynecology | N/A | 382 | 382 | N/A | 28859 | 28859 | N/A | 4135 | 4135 |
| Male genital organs | 1588 | N/A | 1588 | 38206 | N/A | 38206 | 21093 | N/A | 21093 |
| Thyroid | 166 | 399 | 565 | 23719 | 60872 | 84592 | 2306 | 4565 | 6927 |
| Non-Hodgkin's lymphoma | 225 | 162 | 386 | 7193 | 6099 | 13292 | 2214 | 1430 | 3573 |
| Multiple myeloma | 216 | 128 | 343 | 3346 | 2569 | 5915 | 1238 | 779 | 1976 |
| Leukemia | 280 | 193 | 473 | 9286 | 7064 | 16349 | 2475 | 1639 | 4037 |
| CNS | 38 | 15 | 52 | 2282 | 1934 | 4216 | 503 | 327 | 814 |

The numbers of non-AF subjects were adjusted according to the age and sex distribution of AF patients.

* Age adjusted; § Age and sex adjusted

AF, atrial fibrillation; CNS, central nervous system.

**Table S3 Characteristics of study population.**

| Study population | Overall, n | Men, n (%) | Women, n (%) | Age, median (IQR) |
| --- | --- | --- | --- | --- |
| AF and cancer | 22185 | 13631 (61.44%) | 8554 (38.56%) | 77 (67-82) |
| Non-AF and cancer | 839864 | 419020 (49.89%) | 420844 (50.11%) | 67 (57-72) |
| AF and non-cancer | 246216 | 118063 (47.95%) | 128153 (52.05%) | 77 (67-87) |
| Non-AF and non-cancer | 24856182 | 12098872 (48.68%) | 12757310 (51.32%) | 47 (32-62) |
| All | 25964447 | 12649586 (48.72%) | 13314861 (51.28%) | 47 (32-62) |

AF, atrial fibrillation; IQR, interquartile range.

**Figure S1. Similar peak age of specific cancer types between AF and non-AF populations**


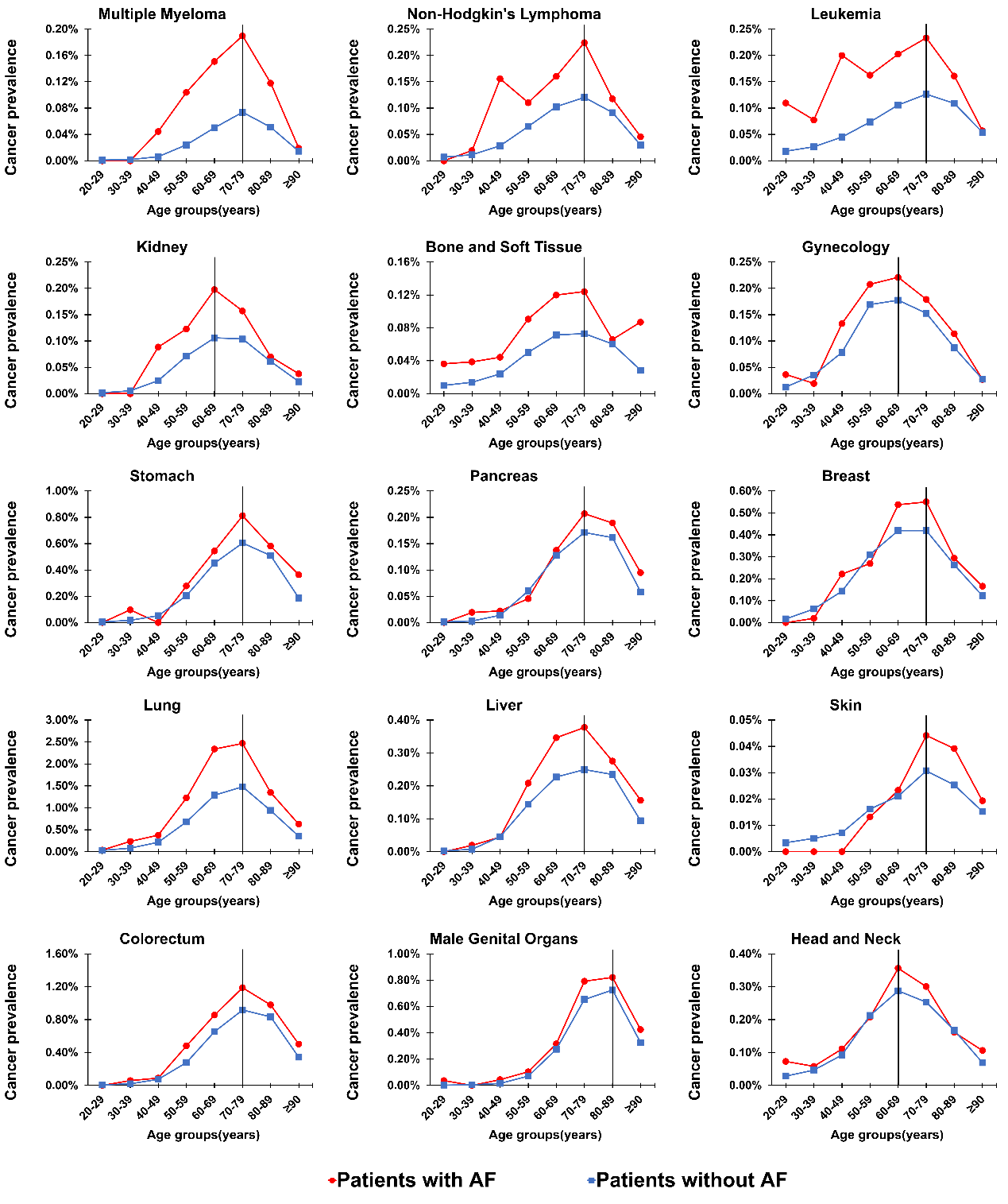


AF, atrial fibrillation.
